# Behavioral Interventions on Periodontitis Patients to Improve Oral Hygiene: A Systematic Review

**DOI:** 10.3390/jcm12062276

**Published:** 2023-03-15

**Authors:** Maria Vilar Doceda, Catherine Petit, Olivier Huck

**Affiliations:** 1 Dental Faculty, University of Strasbourg, 67000 Strasbourg, France; 2 Pole de Médecine et Chirurgie Bucco-Dentaire, Periodontology, Hôpitaux Universitaires de Strasbourg, 67000 Strasbourg, France

**Keywords:** periodontal disease, oral hygiene, psychological intervention, behavioral changes

## Abstract

This systematic review aimed to investigate the impact of different psychological models, strategies, and methods to improve plaque control and/or gingival inflammation in patients with periodontal diseases. Methods: The PubMed/MEDLINE, Cochrane Library, and Embase online databases were explored to identify relevant studies published before October 2022. Articles investigating the effects of different psychological approaches and intervention strategies on periodontitis patients’ oral hygiene (OH) behavioral change were screened. Results: 5460 articles were identified, and 21 fulfilled the inclusion criteria. In total, 2 studies tested audio-visual modalities, and the remaining 19 publications involved six psychological models of health-related behavioral interventions, including Social Cognitive Theory, the Theory of Planned Behavior, the Health Action Process Approach, Leventhal’s self-regulatory theory, Motivational Interviewing, and Cognitive Behavioral Therapy. A meta-analysis of the results was not carried out due to the high heterogeneity among the interventions. Conclusions: Considering the limitations of the available studies, psychological interventions based on social cognitive models that combine some of the techniques of this model (goal setting, planning, self-monitoring, and feedback) may improve OH in periodontitis patients, having a positive impact on periodontal clinical outcomes. Delivering cognitive behavioral therapy in combination with motivational interviewing may result in an improvement in OH as evaluated by decreasing plaque and bleeding scores.

## 1. Introduction

Poor oral hygiene (OH) has been demonstrated to be a major risk factor for periodontal disease development [1]. Indeed, dental plaque has been proven to initiate and promote gingival inflammation, which is a risk factor for further periodontal attachment and tooth loss [2]. Repeated detection of bleeding in probing (BoP) at the same site during supportive periodontal therapy (SPT) was found to be a positive predictive value for subsequent attachment loss [3]. Consequently, patients’ compliance with proper dental hygiene and frequent follow-ups during SPT are all critical to the long-term effectiveness of periodontal therapy [4]. To this end, strategies to boost patient motivation should be included in periodontal therapy.

Since the 1960s, a wide range of psychological models and theories have been developed to positively impact health-related behavioral changes. Social cognition models (SCMs) are based on how people make sense of other people (person perception) and themselves (self-regulation) in order to coordinate with their social world [5,6]. Two broad types of SCMs have been applied in health psychology. The first type focuses on how people respond to a serious illness. Leventhal’s self-regulation model falls into this category, representing the illness in five major dimensions: identity, timeline, illness consequences, risk factors of the disease, and potential for cure or control [7]. The second type of SCMs focuses on different components of an individual’s cognition to anticipate future health-related behaviors [8]. This category includes, among others, the Health Belief Model [9,10], the Theory of Reasoned Action [11], the Theory of Planned Behavior [12], the Social Cognitive Theory [13], and the Health Action Process Approach [14]. Cognitive behavioral therapy (CBT) assumes that “people’s emotions and behaviors are influenced by their perceptions of events” [15,16]. This model is frequently used as the main treatment option for common mental health disorders [16]. Other health behaviors and conditions could be addressed with motivational interviewing (MI), which is intended to increase personal motivation for and commitment to a given goal by analyzing the individual’s own reasons for the change in a compassionate and accepting environment [17] This psychological approach was first developed for the study of addiction and is now widely used to enhance healthy behaviors such as diet, physical exercise, diabetes control, pain management, screening, and medical adherence [18,19,20,21,22,23,24]. 

Until now, narrative reviews have provided a description of specific interventions based on psychological models and theories on OH adherence to propose a framework for increasing our understanding of the determinants of adherence to recommendations concerning health behaviors [25,26,27]. In a recent systematic review and meta-analysis, Carra et al. concluded that psychological interventions based on cognitive constructs and MI may reinforce OH in patients with gingivitis or periodontitis [28]. However, the quantitative analysis of the results failed to demonstrate a significant difference between the groups as measured by the reduction in plaque and bleeding scores over time.

The purpose of this systematic review is to evaluate the impact of various psychological strategies and methods on OH-related behavior changes as reflected by plaque and/or gingival inflammation indices in patients with periodontitis but not gingivitis. To this end, the following review question was formulated: What is the effect of psychological interventions and the OH instructional mode on improving periodontal clinical parameters (plaque and bleeding indices) in periodontitis patients?

## 2. Methods

### 2.1. Search Strategy

This systematic review was conducted according to PRISMA guidelines. To identify relevant studies, three databases: the PubMed/MEDLINE, Cochrane Library, and Embase online databases were searched by two different blinded researchers (M.V.D and O.H) up to October 2022. The following keywords were included and used in various combinations: “periodontitis” OR “periodontal disease (s)” AND “oral hygiene”, “oral hygiene instructions”, “text messaging”, “mobile app”, “intraoral camera”, “plaque disclosing”, “video”, “personalized oral hygiene”, “self-inspection plaque”, “computer”, “phone”, “plaque control”, “teledentistry”, and “psychological oral hygiene”. Each researcher independently selected and reviewed the articles for the inclusion criteria and made a joint decision in case of disagreement.

### 2.2. Inclusion and Exclusion Criteria

The criteria for eligibility for selection of a paper were adult patients (>18 years), with periodontitis, and receiving OH instructional strategies or educational methods to improve OH. Only randomized controlled clinical trials (RCTs), non-randomized controlled clinical trials (NRCTs), cohort studies, and case-control studies with a follow-up of at least 1 month were included. The exclusion criteria for this review were patients who only had gingivitis or patients with comorbidities affecting periodontal status. Additionally, patients with orthodontic appliances were excluded. 

### 2.3. Eligibility Criteria

The following PICOS framework was used to create this systematic review:

- Participants: Adult patients aged 18 or over with periodontitis, excluding patients who only had gingivitis, patients with comorbidities affecting periodontal status (e.g., diabetes mellitus), or patients with orthodontic appliances.
- Interventions: OH instructional strategies and behavioral or educational interventions provided by oral health professionals and/or psychologists/counselors to increase adherence to OH advice.
- Comparison: No OH instructions (OHI) or regular OHI provided by oral health specialists.
- Outcome measures: Any established index for measuring the amount of plaque and inflammation (bleeding before and after the intervention).
- Study design: RCTs, NRCTs, cohort studies, and case-control studies with a follow-up of at least 1 month.

### 2.4. Data Collection

According to the study design, the following parameters collected from the articles were assessed:

- Periodontal status, age, and sample size;
- Study design;
- Type of intervention;
- Follow-up period;
- Measures of periodontal status;
- Impact of interventions on periodontal status.

### 2.5. Quality Assessment

Two researchers (M.V.D and O.H) evaluated the risk of bias independently, using the revised Cochrane risk-of-bias tool for RCTs (RoB-2) [29] and the Newcastle Ottawa Quality Assessment Scale [30] for NRCTs. Disagreements between the researchers were discussed until a consensus was reached.

### 2.6. Synthesis of the Results

A high degree of heterogeneity in the included studies was observed during the initial protocol writing of this systematic review due to the different protocols of interventions as well as the different outcomes measured and follow-up times of reevaluation. It was therefore decided not to pool individual data in a meta-analysis but to perform a narrative overview of the studies.

## 3. Results

### 3.1. Study Selection

**Figure 1:**
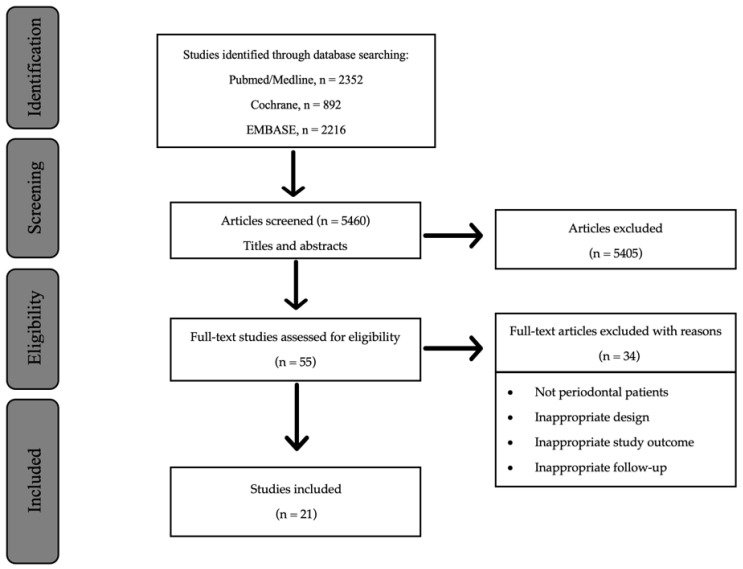
Flowchart showing PRISMA diagram for literature search and inclusion.

After the initial search, 5460 articles were identified. Figure 1 shows the study selection process: 5405 articles were excluded after the screening of titles and abstracts, and 55 were full-text examined. Of these, 34 were removed due to the non-eligible study population, wrong study outcome, inadequate study design, or insufficient follow-up time, based on our inclusion criteria. The summary of the excluded studies [31,32,33,34,35,36,37,38,39,40,41,42,43,44,45,46,47,48,49,50,51,52,53,54,55,56,57,58,59,60,61,62,63,64], as well as the reasons for their exclusion, are listed in Supplementary Materials Table S1. Finally, 21 studies were included in this systematic review.

### 3.2. Study Characteristics

Among the 21 selected studies, 16 were RCT [65,66,67,68,69,70,71,72,73,74,75,76,77,78,79,80], and 5 were NRCTs [81,82,83,84,85] published before October 2022. The sample sizes ranged from 20 to 297 people, with follow-up intervals ranging from 1 month to 3 years. Only adult periodontitis patients were included, ranging in age from 18 to 80 years.

In total, 2 studies tested audio-visual modalities, such as video tapes or PowerPoint presentations, to improve OH habits, and the remaining 19 publications involved six psychological models of health-related behavioral interventions, including Social Cognitive Theory, the Theory of Planned Behavior, the Health Action Process Approach, Leventhal’s self-regulatory theory, MI, and CBT. In one of them, SMS messages were tested as a trigger for changing patients’ behavior and improving periodontal clinical parameters. A wide range of periodontal measures was employed in the listed studies, including any validated plaque and bleeding scores. Table 1, Table 2, Table 3 and Table 4 show the study characteristics based on the type of intervention used.

### 3.3. Risk of Bias of the Included Studies

The overall risk of bias was evaluated by two different authors (M.V.D and O.H) and represented in two separate tables for RCTs (Table 5) and NRCTs (Table 6). Among the NRCTs, a low risk of bias was observed, whereas 12 out of 16 RCTs showed a high risk of bias.

### 3.4. Impact of the Different Strategies Based on Audio-Visual Tools for OHI

Two of the included studies explored the impact of audio-visual tools on improving OH adherence. Williams et al. found that revising the same standard OHI and time for instruction via a power-point presentation versus a personal visit and explanations by a self-care instructor resulted in a significant improvement in plaque scores at a 1-month reevaluation in the young population (<50 years old) and had no positive impact on the older population [65]. However, in a limited sample size of 24 patients, reinforcing OHI with a 12-min videotape at 3 weeks after the initial OH did not produce additional benefits in periodontal clinical outcomes at 2 months [81].

### 3.5. Impact of the Psychological Models of Health-Related Behavior

Social cognitive theory: teaching self-monitoring of the periodontal status and providing feedback on the clinical improvements and positive reinforcement were all part of the social learning interventions. Compared to standard OHI, protocols including behavioral self-inspection training and verbal feedback significantly reduced plaque scores at 2 months [67], 3 months [83], and 4 months of follow-up [66], as well as bleeding on probing [66,83]. Nevertheless, the positive impact of these strategies declined at 7 and 13 months of reevaluation [83]. All of these studies had one thing in common: a high frequency of interventions during the first few weeks of the studies prior to the final outcome. In a RCT, the additional oral self-inspection manual to guide the patients to score the presence of plaque did not appear to demonstrate a significant improvement in plaque and bleeding scores compared to a traditional group of experimental instructions delivered by an experienced dental hygienist at any time up to 6 months of reevaluation [68]. Glavind et al. reported similar findings in two RCTs with a 6-month follow-up when a written self-instructional manual of OHI was compared to regular OHI as a control group [84] as well as a group in which personalized OHI was provided [82]. 

Asimakopoulou et al. evaluated the impact of combining several social learning elements on behavioral changes and periodontal clinical indicators, including customized risk communication, goal-setting, self-monitoring, and planning, in 97 individuals. At one and three months, individualized risk communication alone or in combination with setting goals, self-monitoring, and planning outperformed the control group significantly [79]. 

Theory of reasoned action: Jönsson and colleagues extracted the data reported in a RCT [77] to test the direct and indirect pathways within the extended TRA model in 113 subjects. The extended TRA model explained a significant amount of variation in gingival outcome scores (56%) after 12 months. At three months, having a higher level of self-efficacy at baseline was coupled with achieving a higher frequency of OH behavior [85]. 

Motivational interviewing and Leventhal’s self-regulatory theory: The impact of MI on periodontal clinical outcomes was studied in four RCTs. High heterogeneity was observed in terms of the time and number of the interventions, as well as the people who were trained to do them. In two separate publications of the same study’s short and long-term outcomes, a clinically experienced psychologist delivered one 20–90 min session of MI [69,70]. In another RCT, a trained and experienced counselor in MI delivered a brief session of 15–20′ [71], whereas trained dental students were responsible for 4–5 sessions of MI interventions of periodontitis patients prior to periodontal therapy [72]. Despite the different approaches used in the previous studies, no statistically significant results were observed when compared to the control groups’ conventional OHI. Godard et al. aimed to see if MI covering Leventhal’s five dimensions outperformed traditional basic training in terms of enhancing plaque control compliance among periodontitis patients. Patients in the experimental group exhibited considerably improved OH after 1 month of follow-up [73]. Philippot and colleagues also investigated if Leventhal’s autoregulation hypothesis may enhance periodontitis patients’ compliance during a one-month follow-up. In this short-term RCT of 30 patients, the experimental group had significantly lower plaque scores on the proximal and lingual sides than the control group [74]. 

Cognitive behavioral therapy: Four RCTs were categorized as CBT among the included studies, and three of them were conducted by the same research group [76,77,78]. In summary, 3 months after executing four sessions of the Client Self-Care Commitment Model (CSCCM) by an experienced dental hygienist versus three sessions of standard OHI, Jönsson and coworkers revealed a statistically significant improvement in the plaque index and interdental cleaning [76]. In 2009, the same group published a separate trial using a different approach for the test group and a larger sample size of 113 patients [77]. These data were also utilized in a third publication [78] with different study outcomes. In both trials [77,78], a trained dental hygienist offered 5–9 extended visits of an individually tailored oral health educational program based on cognitive behavioral principles using MI methods. The patients in the control group underwent routine periodontal treatments. As a result, all clinical indices improved significantly after three months and remained stable after a year [77,78]. 

In a long-term RCT, 26 patients were included to examine the psychological blocks of unmotivated periodontitis patients using an exploratory listening technique by a psychologist. The authors concluded that there was a significant treatment effect in the test group whose PI improved to below 50% after 1 year, whereas, in group C (control), the majority of patients remained stable or worsened [75]. 

Mobile text messages and Health Action Process Approach: Only one RCT investigated the additional impact of receiving mobile text messages on the reinforcement of OH compliance. When compared to a control group that did not receive text messages, this tool appeared to dramatically improve bleeding ratings in the test group after 4 months of follow-up. Only intention and recovery self-efficacy modestly increased with the use of text messages when assessing the influence of the intervention on the psychological determinants of OH [80].

## 4. Discussion

The evidence provided in this systematic review highlighted a potential benefit of combining some of the principles included in social learning theory, such as goal setting, planning, self-monitoring, and feedback [66,67,79,83]. MI alone did not significantly outperform plaque and bleeding scores in long-term clinical trials [69,70,71,72]; however, delivering individually tailored oral health education programs based on cognitive behavioral principles in combination with MI may result in an improvement in OH as evaluated by decreasing plaque and bleeding scores, according to the research examined [77,78]. These statements are consistent with the 11th European Workshop recommendations on the use of psychological interventions (goal setting, planning, self-monitoring) to improve OH-related behaviors in periodontal disease patients [86,87]. In addition, Carra et al. suggested that psychological interventions based on cognitive models and MI may reinforce OH in periodontitis patients [28]. 

It should be mentioned that the studies that were chosen employed a range of validated measures to quantify the level of plaque and gingival inflammation, and some of them did not investigate both clinical outcomes at the same time. This circumstance may hamper the data comparability within the studies.

The duration of the studies and the frequency of the interventions are also important considerations in evaluating the efficacy of behavioral interventions. Old studies have already highlighted the significance of repeating OHI at regular intervals to compensate for the relapse of new OH habits over time [82], which may help avoid recurrent periodontitis [68]. Different follow-up periods, ranging from 1 month to 3 years, were observed in this systematic review. This is a disadvantage in terms of interpreting and comparing the data. In three of the reviewed studies, the authors only conducted a one-month follow-up [65,73,74]. Two of them exclusively employed plaque scores as an outcome measure to investigate the impact of Leventhal’s self-regulatory theory on periodontitis patients’ OH behavioral changes [73,74], revealing a significant clinical improvement in this parameter. When compared to the control group, Williams et al. found no significant clinical improvements in plaque and bleeding scores after one month of follow-up [65]. Long-term studies are thus required to minimize the bias of periodontal treatment’s positive short-term effects and to properly explore the long-term influence of psychological models and OH instructional strategies on plaque reduction and, consequently, bleeding scores. 

Behavioral and psychological interventions have been traditionally implemented for smoking cessation [88], to prevent some chronic diseases, and to cope with symptoms in patients who have been medically treated for cancer, cardiovascular disease, or HIV/AIDS [89]. Moreover, the increased usage of mobile technology is enabling new and innovative approaches to healthcare delivery [90]. In a recent systematic review and meta-analysis, the use of smartphone apps, text messages, and computer-aided learning for improving OH resulted in a large reduction in the plaque and gingival index [91]. These strategies seem to be promising clinical tools for children and young people who are highly attracted to and widely accustomed to these devices. In line with these publications, a post hoc analysis by age showed significant improvement in the plaque score in young participants trained in OH with a PowerPoint presentation compared to a self-care instructor in a short-term study [65]. Additionally, these technologies can be useful for consolidating newly learned behaviors over time. Araújo et al. demonstrated significant improvement in BOMP in a 4-month RCT by using weekly text messages related to OH and gingival inflammation [80]. 

In order to make the different interventions used more understandable, the authors of this systematic review opted to group the research according to the currently reported behavioral and psychological therapies. However, the work was complicated by the overlap of different approaches utilized in some of the research, as well as the absence of precise definitions and taxonomy for the treatments. For example, Asimakopoulou et al. explored for the first time the combined effect of goal setting, planning, and self-monitoring with risk communication practice in a recent RCT [79]. In another study, Godard et al. delivered 15–20 min of a single session of MI guided by Leventhal’s self-regulatory theory [73]. However, the spirit of MI is to elicit information from the patient rather than impart it [17], which appears to be at odds with Leventhal’s educational nature which provides information about the disease in order to motivate patients to cure and control the illness [7]. A similar example was observed in another RCT [77], which included methods of MI for cognitive behavioral interventions, with the unclear specification of the protocols applied [86].

## 5. Limitations

Overall, the scientific evidence from the studies included in this systematic review was not strong due to the short follow-up times, the small sample sizes, and the lack of clear or confusing randomization methods. Among the RCTs, at least 12 articles, potentially 16, showed a high risk of bias. Furthermore, due to the significant heterogeneity of the included research, a quantitative analysis of the outcomes was unfeasible, hence, only a summary of the studies was possible. 

The heterogeneity of the studies was reflected in the different durations of the studies, clinical measures obtained, frequency of interventions, and protocols applied to the interventions. In addition to attempting to compare a wide range of psychological interventions and learning methodologies, the studies categorized according to the same psychological model were very different from each other. The studies that used MI are a good example of this. We noticed a dearth of specific information on the types of interventions and procedures utilized in this research. Furthermore, the quality of the interventions was only evaluated in three trials [69,70,72] to guarantee that all the elements of MI were met. Another key consideration is the professional’s expertise, training, and background in delivering MI (caregivers, psychologists, counselors, periodontists, or dental students). This element may have an impact on not only the quality of the intervention but also the patient’s acceptance and the two parties’ trust relationship. For the reasons stated above, as well as the nature of the interventions, there is an inherent risk of bias in the evaluation of the results.

## 6. Conclusions

Considering the limitations of the available studies, psychological interventions based on social cognitive models that combine some of the techniques of this model (goal setting, planning, self-monitoring, and feedback) may improve OH in periodontitis patients and have a positive impact on periodontal clinical outcomes. Additionally, offering CBT in combination with MI approaches may result in an improvement in OH, as measured by plaque and bleeding scores. Nevertheless, due to the limitations of attempting to compare such different research, these results should be interpreted with caution.

For future studies, a more precise categorization of the psychological interventions applied, based on standardized vocabulary to define the intervention components, seems to be necessary [92]. A clear and detailed description of the content may ensure the fidelity in replication of the interventions [93]. Additionally, the standardization of training for counselors could also improve the studies’ reproducibility, improving homogeneity and facilitating the analysis of the results.

## Figures and Tables

**Table 1:** Description of the included studies exploring the impact of the OH instructional mode on the behavior and periodontal status of patients with periodontal disease.

Reference	Periodontal Status Age (Year) Sample Size	Study Design	Intervention	Follow-Up	Outcome Assessed	Impact on Plaque Score {Mean (SD)} or {Percentage %}	Impact on Bleeding Score {Mean (SD)} or {Percentage %}
AUDIO-VISUAL POWERPOINT							
Williams et al. 2018 [65]	Mild to moderate periodontitis (PD < 6 mm) 21–80 years *n* = 58 T group *n* = 30 C group *n* = 28	RCT	Same OHI delivered: Test: Computer-teaching format (8 min audio-visual PowerPoint presentation containing 12 slides). Control: Self-care instructor (8 min).	Baseline (T0) 4 weeks (T1)	6 tooth surfaces: PS (O’Leary) BI (Silness and Loe) BoP%	TEST Baseline: 68 ± 10.7 At 4 weeks: 79.8 ± 11.4 CONTROL Baseline: 65.8 ± 7.1 At 4 weeks: 76.5 ± 11.9	TEST Baseline: 0.28 ± 0.1 42% ± 15.3 At 4 weeks: 0.23 ± 0.09 32.2% ± 20.9 CONTROL Baseline: 0.26 ± 0.1 37.8% ± 15.2 At 4 weeks: 0.17 ± 0.1 30.6% ± 10.7
VIDEOTAPE							
Glavind et al., 1986 [81]	Few periodontal pockets > 5 mm 32–63 years *n* = 24 T group *n* = 12 C group *n* = 12	NRCT	Both groups: OHI. Test: Reinforcement of the OHI by videotape (12 min) at the 3-week follow-up.	Baseline (T0) 2 weeks (T1) 3 weeks (T2) 8 weeks (T3)	4 tooth surfaces: PI%: (presence/ absence) BI%: (presence/ absence)	TEST Baseline: 62% (16.8) At 2 weeks: 59% (16.7) At 3 weeks: 29% (19.5) At 8 weeks: 28% (16.3) CONTROL Baseline: 58% (16.2) At 2 weeks: 52% (18.4) At 3 weeks: 23% (19.0) At 8 weeks: 22% (12.5)	TEST Baseline: 51% (19.8) At 8 weeks: 29% (17.0) CONTROL Baseline: 45% (14.8) At 8 weeks: 24% (14.8)

Abbreviations: RCT, randomized clinical trial; NRCT, non-randomized clinical trial; T, test; C, control; OHI, oral hygiene instructions; PD, pocket depth; PPD, probing pocket depth; PI, plaque index; PS, plaque score; BoP, bleeding on probing; and BI, bleeding index.

**Table 2:** Summary of the strategy and results of the included studies exploring the impact of OH instructional mode on the behavior and periodontal status of patients with periodontal disease.

Reference	Strategy	Results
Williams et al., 2018 [65]	OHI given in a computer-assisted format (PowerPoint presentation).	No statistically significant difference between the groups. PLAQUE: Significant differences between older and younger participants (<50 years old) trained on the computer. Younger sample was significantly better using the computer format.
Glavind et al., 1986 [81]	Reinforcement of the OHI by showing a television tape.	No statistically significant difference between the groups.

**Table 3:** General information of the included studies exploring the impact of psychological models of health-related behavior on the behavior and periodontal status of patients with periodontal disease.

Reference	Periodontal Status Age (Year) Sample Size	Study Design	Intervention	Follow-Up	Outcome Assessed	Impact on Plaque Score {Mean (SD)} or {Percentage %}	Impact on Bleeding Score {Mean (SD)} or {Percentage %}
SOCIAL COGNITIVE THEORY							
Little et al., 1997 [66]	Mild to moderate periodontal disease (at least 6 sites PD 4–7 mm) 50–70 years *n* = 107 T group: *n* = 54 C group: *n* = 53	RCT	Test: 5 weekly, 90-min sessions: skill training, self-monitoring, and feedback Control: Usual periodontal maintenance care	Baseline (T0) 4 months (T1)	4 tooth surfaces: PI (O’Leary) GI BoP (%) PPD CAL	TEST Baseline: 82% At 4 months: 76% CONTROL Baseline: 80% At 4 months: 80%	TEST Baseline: 24% At 4 months: 15% CONTROL Baseline: 26% At 4 months: 21%
Weinstein et al., 1996 [67]	Periodontitis patients 32–50 years *n* = 20 Control group 1: *n* = 5 Control group 2: *n* = 5 Test group 1: *n* = 5 Test group 2: *n* = 5	RCT	Control 1: Bass technique Control 2: Bass technique + 2× weekly verbal feedback Test 1: Bass technique + 2× weekly verbal feedback + positive reinforcement Test 2: Bass technique + 2× weekly verbal feedback + positive reinforcement + Self-monitoring	Baseline (T0) 1 month (T1) 2 months (T2)	FMPS (O’Leary)	CONTROL 1 Baseline: 0.397 (0.165) At 1 month: 0.390 (0.175) At 2 months: 0.384 (0.159) CONTROL 2 Baseline: 0.395 (0.086) At 1 month: 0.271 (0.096) At 2 months: 0.323 (0.079) TEST 1 Baseline: 0.353 (0.187) At 1 month: 0.205 (0.091) At 2 months: 0.228 (0.075) TEST 2 Baseline: 0.376 (0.058) At 1 month: 0.121 (0.017) At 2 months: 0.148 (0.034)	NR
Baab et al., 1986 [68]	Periodontitis patients who had completed active periodontal treatment 30–76 years, *n* = 31 T group *n* = 15 C group *n* = 16	RCT	Both groups: OHI. Test: oral self-inspection manual	Baseline (T0) 2 weeks (T1) 1.5 months (T2) 3 months (T3) 6 months (T4)	6 tooth surfaces: Plaque% (O’Leary) Gingival bleeding%	Outcomes of measurements are not described numerically	Outcomes of measurements are not described numerically
Glavind et al., 1981 [82]	Few periodontal pockets > 5 mm 25–64 years, *n* = 37 Group 1 *n* = 12 Group 2 *n* = 13 Group 3 *n* = 12	NRCT	Group 1: Written self-instructional manual of OH Group 2: Individualized OHI Group 3: Minimal OHI	Baseline (T0) 1 week (T1) 2 weeks (T2) 6 weeks (T3) 3 months (T4) 6 months (T5)	4 tooth surfaces: PI%: (presence/ absence) BI%: (presence/ absence)	GROUP 1 Baseline: 66.2 (19.7) At 1 week: 44.1 (17.2) At 2 weeks: 22.3 (18.7) At 6 weeks: 21.5 (20.4) At 3 months: 17.2 (14.2) At 6 months: 20.4 (15.9) GROUP 2 Baseline: 61.4 (19.3) At 1 week: 43.8 (20.3) At 2 weeks: 27.5 (20.9) At 6 weeks: 23.3 (19.1) At 3 months: 25.1 (21.3) At 6 months: 22.1 (19.2) GROUP 3 Baseline: 66.1 (16.7) At 1 week: 48.1 (16.6) At 2 weeks: 25.6 (16.8) At 6 weeks: 26.4 (20.7) At 3 months: 19.6 (12.0) At 6 months: 19.7 (15.9)	GROUP 1 Baseline: 39.5 (24.4) At 6 weeks: 14.1 (15.7) At 3 months: 18.0 (16.3) At 6 months: 13.1 (14.8) GROUP 2 Baseline: 39.6 (26.9) At 6 weeks: 15.2 (17.6) At 3 months: 18.0 (15.2) At 6 months: 13.1 (10.6) GROUP 3 Baseline: 39.6 (20.9) At 6 weeks: 15.0 (13.6) At 3 months: 14.5 (14.9) At 6 months: 15.9 (12.9)
Glavind et al., 1983 [83]	Few periodontal pockets > 5 mm 22–67 years, *n* = 63 Group B: brushing test *n* = 17 Group O: open scoring *n* = 14 Group M: minimal feedback *n* = 17 Group C: control *n* = 15	NRCT	Group B: Written self-instructional manual of OH + feedback + “tooth brushing test”. Group O: Written self-instructional manual of OH + feedback Group M: Written self-instructional manual of OH Group C: Minimal OHI	Baseline (T0) 1 week (T1) 2 weeks (T2) 6 weeks (T3) 3 months (T4) 7 months (T5) 13 months (T6)	4 tooth surfaces: PI%: (presence/ absence) BI%: (presence/ absence)	GROUP B Baseline: 60.9 (19.6) At 1 week: 35.6 (11.9) At 2 weeks: 23.1 (14.8) At 6 weeks: 28.5 (16.3) At 3 months: 26.5 (18.0) At 7 months: 37.5 (14.5) At 13 months: 35.7 (16.4) GROUP O Baseline: 62.8 (17.2) At 1 week: 37.2 (17.5) At 2 weeks: 27.1 (20.3) At 6 weeks: 27.1 (14.7) At 3 months: 22.4 (18.8) At 7 months: 31.8 (16.6) At 13 months: 30.4 (19.3) GROUP M Baseline: 61.9 (18.3) At 1 week: 36.4 (20.6) At 3 months: 34.4 (21.3) At 7 months: 33.3 (21.0) At 13 months: 29.9 (13.9) GROUP C Baseline: 62.0 (16.8) At 1 week: 34.5 (12.7) At 3 months: 35.3 (12.6) At 7 months: 34.3 (15.6) At 13 months: 37.0 (15.7)	GROUP B Baseline: 49.0 (21.9) At 6 weeks: 29.5 (16.9) At 3 months: 17.2 (14.8) At 7 months: 24.3 (13.5) At 13 months: 24.0 (17.6) GROUP O Baseline: 54.5 (18.3) At 6 weeks: 28.6 (17.3) At 3 months: 13.9 (12.4) At 7 months: 20.9 (15.5) At 13 months: 19.6 (16.4) GROUP M Baseline: 50.3 (16.7) At 6 weeks: 29.8 (14.5) At 3 months: 13.6 (12.1) At 7 months: 22.0 (20.2) At 13 months: 19.9 (13.8) GROUP C Baseline: 53.6 (23.5) At 6 weeks: 30.1 (14.6) At 3 months: 18.8 (11.7) At 7 months: 22.5 (14.4) At 13 months: 34.8 (15.9)
Glavind et al., 1984 [84]	Few periodontal pockets > 5 mm 22–67 years, *n* = 74 Group 1 *n* = 23 Group 2 *n* = 27 Group 3 *n* = 24	NRCT	Group 1: Self-examination prior to OHI Group 2: OHI Group 3: Delayed OHI (at 6 weeks)	Baseline (T0) 1 week (T1) 2 weeks (T2) 6 weeks (T3) 7 weeks (T4) 3 months (T5) 7 months (T6)	4 tooth surfaces: PI%: (presence/ absence) BI%: (presence/ absence)	GROUP 1 Baseline: 61.8 (15.7) At 2 weeks: 40.7 (17.4) At 6 weeks: 38.3 (21.0) At 3 months: 31.5 (21.3) At 7 months: 23.7 (16.8) GROUP 2 Baseline 59.4 (17.0) At 2 weeks: 44.3 (17.3) At 6 weeks: 34.3 (16.7) At 3 months: 30.1 (17.2) At 7 months: 23.5 (14.9) GROUP 3 Baseline: 60.3 (16.8) At 6 weeks: 52.0 (17.7) At 7 weeks: 24.5 (13.9) At 3 months: 27.1 (20.3) At 7 months: 19.7 (16.8)	GROUP 1 Baseline: 55.4 (14.4) At 6 weeks: 33.3 (18.5) At 3 months: 17.3 (11.4) At 7 months: 20.4 (11.8) GROUP 2 Baseline: 52.6 (19.2) At 6 weeks: 31.4 (16.8) At 3 months: 19.2 (14.4) At 7 months: 21.9 (13.9) GROUP 3 Baseline: 56.3 (21.2) At 6 weeks: 45.7 (18.9) At 3 months: 21.4 (18.4) At 7 months: 18.0 (17.1)
RISK COMMUNICATION, GOAL SETTING, PLANNING, AND SELF-MONITORING							
Asimakopoulou et al., 2019 [79]	Periodontitis patients Mean age: 60.61 (11.24) *n* = 97 T group 1 (RISK) *n* = 32 T group 2 (GPS) *n* = 33 C group (TAU) *n* = 32	RCT	All groups: OHI T Group 1: 5–10′ explanation of their individualized risk T Group 2: 5–10′ explanation of their individualized risk + setting goals, self-monitoring, and planning	Baseline (T0) 1 month (T1) 3 months (T2)	4 tooth surfaces: PI% (presence/ absence) 6 tooth surfaces: BoP% (presence/ absence) PPD	TEST 1 (RISK) Baseline: 21.59% (15.49) At 1 month: 12.21% (9.33) At 3 months: 9.87% (7.93) TEST 2 (GPS) Baseline: 16.23% (10.54) At 1 month: 10.91% (9.90) At 3 months: 9.65% (8.06) CONTROL (TAU) Baseline: 13.97% (10.30) At 1 month: 10.87% (7.22) At 3 months: 10.60% (7.66)	TEST 1 (RISK) Baseline: 13.89% (14.88) At 1 month: 5.44% (6.40) At 3 months: 6.72% (7.03) TEST 2 (GPS) Baseline: 9.94% (7.33) At 1 month: 6.11% (7.80) At 3 months: 4.42% (4.23) CONTROL (TAU) Baseline: 8.62% (6.13) At 1 month: 4.37% (3.64) At 3 months: 4.17% (5.51)
THEORY OF REASONED ACTION							
Jönsson et al., 2012 [85]	Moderate to advanced periodontitis and PI > 0.3 Mean age: T = 52.4 (8.4) C = 50.1 (10.3) *n* = 113 T group *n* = 57 C group *n* = 56	Data from RCT (Jönsson et al., 2009, 2010)	Questionnaire: Theory of Reasoned Action	Baseline (T0) 3 months (T1) 12 months (T2)	NR	NR	NR
TEXT MESSAGES AND HEALTH ACTION PROCESS APPROACH							
Araújo et al., 2020 [80]	Periodontal pockets > 3 mm ≥18 years, *n* = 142 C group (FF) *n* = 43 T group 1 (NFH) *n* = 38 T group 2 (TM + NFH) *n* = 61	RCT	All groups: HAPA Questionnaire Patient motivation, discussion about treatment needs, goal setting, and individualized OHI (60′) C group: Finger Floss (FF) T group 1: Novel Floss Holder (NFH) T group 2: Novel Floss Holder + Text Messages (TM + NFH)	Baseline (T0) 4 months (T1)	Bleeding on Marginal Probing index (BOMP)	NR	TEST 1 (NFH) Baseline: 1.14 At 4 months: 0.81 TEST 2 (TM + NFH) Baseline: 1.19 At 4 months: 0.62 CONTROL (FF) Baseline: 1.15 At 4 months: 0.82
LEVENTHAL’S SELF-REGULATORY THEORY							
Philippot et al., 2005 [74]	Periodontitis patients 20–68 years, *n* = 30 T group: *n* = 15 C group: *n* = 15	RCT	Both groups: Information and training of self-care T group: Leventhal’s theory Daily records of improvements in periodontal symptoms	Baseline (T0) 1 month (T1)	PI (Silness and Löe)	TEST Baseline: Global 1.63 (0.43) Lingual 1.87 (0.49) Buccal 1.13 (0.55) Proximal 1.83 (0.41) At 1 month: Global 0.24 (0.19) Lingual 0.22 (0.28) Buccal 0.08 (0.08) Proximal 0.43 (0.24) CONTROL Baseline: Global 1.88 (0.41) Lingual 2.03 (0.41) Buccal 1.41 (0.64) Proximal 2.19 (0.40) At 1 month: Global 0.88 (0.38) Lingual 0.84 (0.48) Buccal 0.45 (0.43) Proximal 1.34 (0.55)	NR
MOTIVATIONAL INTERVIEWING GUIDED BY LEVENTHAL’S SELF-REGULATORY THEORY							
Godard et al., 2011 [73]	Moderate-to- severe chronic periodontitis Mean age: T = 51.6 (16.6) C = 48.3 (16.5) *n* = 51 T group *n* = 27 C group *n* = 24	RCT	All groups: OHI T group: Single session of MI guided by Leventhal’s theory (15–20′), by 2 experienced periodontists	Baseline (T0) 1 month (T1)	3 tooth surfaces: PI (O’Leary)	TEST Baseline: Lingual 35% (0.23) Buccal 58% (0.28) Proximal 65% (0.22) At 1 month: Lingual 18% (0.20) Buccal 29% (0.29) Proximal 45% (0.30) CONTROL Baseline: Lingual 37% (0.23) Buccal 59% (0.19) Proximal 68% (0.23) At 1 month: Lingual 27% (0.16) Buccal 43% (0.22) Proximal 73% (0.27)	NR
MOTIVATIONAL INTERVIEWING							
Stenman et al., 2012 [69]	Moderate chronic periodontitis Mean age: T = 51.9 (8.9) C = 48.9 (12.1) *n* = 39 T group: *n* = 19 C group: *n* = 20	RCT	All groups: OHI T group: Single session of 20–90′ MI by a psychologist	Baseline (T0) 2 weeks (T1) 4 weeks (T2) 12 weeks (T3) 26 weeks (T4)	6 tooth surfaces: PI (O’Leary) Marginal gingival bleeding (MBI) (%)	TEST Baseline: 50.2% (21.5) At 3 months: 27.1% (15.2) At 6 months: 25.2% (15.4) CONTROL Baseline: 43.1% (19.2) At 3 months: 19% (13.3) At 6 months: 18.6% (13.2)	TEST Baseline: 36.6% (17.1) At 3 months: 21% (12.5) At 6 months: 18.8% (10.9) CONTROL Baseline: 33% (12.4) At 3 months: 16.2% (13.4) At 6 months: 18.4% (14.1)
Stenman et al., 2018 [70]	Moderate chronic periodontitis Mean age: T = 58.3 (10.2) C = 54.2 (10.1) *n* = 26 T group: *n* = 13 C group: *n* = 13	RCT	All groups: OHI T group: Single session of 20–90′ MI by a psychologist	Baseline (T0) 6 months (T1) 3 years (T2)	6 tooth surfaces: PI (O’Leary) Marginal gingival bleeding (MBI) (%)	TEST Baseline: 49.6% (23.7) At 6 months: 25.26% (15.3) At 3 years: 42.1% (30.6) CONTROL Baseline: 38.4% (15.3) At 6 months: 15.7% (10.4) At 3 years: 41.9% (30.3)	TEST Baseline: 37.8% (19.7) At 6 months: 17.1% (8.6) At 3 years: 14.7% (9.2) CONTROL Baseline: 32.1% (12.3) At 6 months: 16.3% (8.9) At 3 years: 15.4% (17.6)
Brand et al., 2013 [71]	Patients in periodontal maintenance for at least one year and with a BOP ≥ 40% or at least two teeth with interproximal PD ≥ 5 mm Mean age: 61.9 (11.0) *n* = 56 T group: *n* = 29 C group: *n* = 27	RCT	All groups: Individualized OHI T group: Single brief session of MI (15–20′) by a trained and experienced counselor in MI	Baseline (T0) 6 weeks (T1) 3 months (T2)	6 tooth surfaces: PI (Quigley–Hein) (Ramfjord teeth) BoP (%) PPD	TEST Baseline: 2.4 (0.6) At 6 weeks: 1.9 (0.6) At 3 months: 2.1 (0.7) CONTROL Baseline: 2.6 (0.5) At 6 weeks: 2.2 (0.4) At 3 months: 2.3 (0.7)	TEST Baseline: 50% (18) At 6 weeks: 31% (14) At 3 months: 33% (15) CONTROL Baseline: 55% (18) At 6 weeks: 40% (19) At 3 months: 36% (20)
Woelber et al., 2016 [72]	CPITN ≥ 3 of at least two sextants Mean age: 59.27 (11.40) *n* = 172 T group: *n* = 73 C group: *n* = 99	RCT	All groups: OHI T group: 4–5 sessions of MI delivered by dental students trained in MI	Baseline (T0) 6 months (T1)	PI (Silness and Löe) GI (Löe and Silness) BoP (%) PPD CAL	TEST Baseline: 0.56 (0.3) At 6 months: 0.72 (0.32) CONTROL Baseline: 0.43 (0.30) At 6 months: 0.54 (0.32)	TEST Baseline: 51.87% (23.18) At 6 months: 46.65% (25.07) CONTROL Baseline: 53.65% (23.86) At 6 months: 51.82% (27.32)
COGNITIVE BEHAVIOURAL THERAPY							
Alcouffe et al., 1988 [75]	Periodontitis patients with no sites of active periodontitis, who did not respond adequately to hygiene instructions (PI > 50%) 29–72 years, *n* = 26 T group: *n* = 13 C group: *n* = 13	RCT	All groups: 4 teaching sessions of OH T group: Interviewed by a psychologist (50–90′): perception of periodontal disease, notions of recovery, prevention, and personal hygiene measures	Baseline (T0) Every 3 months for 2 years	PI (O’Leary)	TEST Baseline: 68.08 (12.06) At 3 months: 55.31 (13.36) At 6 months: 49.0 (22.58) At 1 year: 50. 64 (20.69) At 2 years: 48.7 (22.32) CONTROL Baseline: 69.38 (10.91) At 3 months: 68.77 (12.21) At 6 months: 67.58 (15.97) At 1 year: 66.55 (18.32) At 2 years: 65.80 (20.60)	NR
Jönsson et al., 2006 [76]	Periodontitis patients with insufficient compliance and progress of their periodontal disease Mean age: T = 54.8 (11.7) C = 58.1 (9.9) *n* = 35 T group *n* = 19 C group *n* = 16	RCT	T group: 4 sessions of Client Self-care Commitment Model (CSCCM) by an experienced dental hygienist C group: 3 sessions of conventional OHI	Baseline (T0) 3 months (T1)	6 tooth surfaces: PI (Silness and Löe) GI (Löe and Silness) BoP% (4 tooth surfaces) PPD	TEST Baseline: 0.59 (0.17) At 3 months: 0.25 (0.11) CONTROL Baseline: 0.59 (0.29) At 3 months: 0.33 (0.11)	TEST Baseline: 46.8% (13.8) At 3 months: 18.7% (8.3) CONTROL Baseline: 39% (16.0) At 3 months: 16.3% (5.7)
COGNITIVE BEHAVIOURAL THERAPY + MOTIVATIONAL INTERVIEWING							
Jönsson et al., 2009 [77]	Moderate to advanced periodontitis and PI > 0.3 Mean age: T = 52.4 (8.4) C = 50.1 (10.3) *n* = 113 T group *n* = 57 C group *n* = 56	RCT	T group: 5–9 visits of individually tailored oral health educational program based on CBT, using MI, delivered by trained dental hygienist C group: 4–8 visits of OHI One visit lasts 45 to 60 min	Baseline (T0) 3 months (T1) 12 months (T2)	6 tooth surfaces: PI (Silness and Löe) GI (Löe and Silness) BoP% PPD	TEST Baseline: 0.74 (0.34) At 3 months: 0.17 (0.11) At 12 months: 0.14 (0.13) CONTROL Baseline: 0.73 (0.31) At 3 months: 0.32 (0.22) At 12 months: 0.31 (0.16)	TEST Baseline: 0.92 (0.28) At 3 months: 0.27 (0.14) At 12 months: 0.21 (0.16) CONTROL Baseline: 0.92 (0.23) At 3 months: 0.52 (0.20) At 12 months: 0.50 (0.17)
Jönsson et al., 2010 [78]	Moderate to advanced periodontitis and PI > 0.3 Mean age: T = 52.4 (8.4) C = 50.1 (10.3) *n* = 113 T group *n* = 57 C group *n* = 56	RCT	T group: 5–9 visits of individually tailored oral health educational program based on CBT, using MI, delivered by trained dental hygienist C group: 4–8 visits of OHI One visit lasts 45 to 60 min	Baseline (T0) 3 months (T1) 12 months (T2)	6 tooth surfaces: PI (Silness and Löe, expressed as % plaque scores ≥ 1) BoP% PPD	TEST Baseline: 59% (18) At 3 months: 17% (10) At 12 months: 14% (12) CONTROL Baseline: 57% (17) At 3 months: 28% (17) At 12 months: 28% (13)	TEST Baseline: 70% (20) At 3 months: 24% (12) At 12 months: 19% (13) CONTROL Baseline: 75% (18) At 3 months: 33% (15) At 12 months: 29% (14)

Abbreviations: CI, confidence intervals; OR, odds ratio; NR, not reported; RCT, randomized clinical trial; NRCT, non-randomized clinical trial; T, test; C, control; OHI, oral hygiene instructions; MI, motivational interviewing; PD, pocket depth; PPD, probing pocket depth; PI, plaque index; PS, plaque score; FMPS, full mouth plaque score; GI, gingival index; GB, gingival bleeding; BoP, bleeding on probing; BI, bleeding index; CAL, clinical attachment level; HAPA, Questionnaire about oral health behaviors and psychological assessment; FF, finger floss; NFH, novel floss holder; and MBI, marginal gingival bleeding.

**Table 4:** Summary of the strategy and results of the included studies exploring the impact of psychological models of health-related behavior on the behavior and periodontal status of patients with periodontal disease.

Reference	Strategy	Results
Little et al., 1997 [66]	5 weekly, 90-min sessions including skill training, self-monitoring, and feedback.	Test group: Significantly increased their skills and frequency of tooth brushing and flossing. Significant reduction in PI and BoP. Significant relative improvement in PD reduction in PD 3–6 mm.
Weinstein et al., 1996 [67]	2× weekly verbal feedback, positive social reinforcement, and self-monitoring.	Significant motivation of periodontal patients to conduct the OH routine.
Baab et al., 1986 [68]	Oral hygiene self-inspection manual.	No statistically significant difference between the groups.
Glavind et al., 1981 [82]	Written self-instructional manual of OH and individual OHI.	No statistically significant difference between the groups.
Glavind et al., 1983 [83]	Written self-instructional manual of OH, feedback, and “tooth brushing test”.	At 3 months: the “tooth brushing test” and feedback significantly improved plaque scores compared to the other groups. At 13 months: the control group showed a significantly higher gingival bleeding score than the others.
Glavind et al., 1984 [84]	Self-examination prior to OHI and delayed OHI.	At 6 weeks, the delayed OHI group showed significantly higher plaque and bleeding scores compared to the other groups. At 3 months, no statistically significant difference between the groups was observed.
Asimakopoulou et al., 2019 [79]	5–10′ explanation of the individualized risk, setting goals, self-monitoring, and planning.	Individualized risk assessment, setting goals, self-monitoring, and planning showed a statistically significant reduction in the percentage of plaque at 1 month and 3 months. Significant improvement in interdental cleaning frequency.
Jönsson et al., 2012 [85]	Questionnaire: Theory of Reasoned Action.	Self-efficacy, gender, and cognitive behavioral intervention were important predictors of OH behavioral change.
Araújo et al., 2020 [80]	Questionnaire (HAPA). Patient motivation, desired outcomes, treatment needs, goal setting, individualized OHI, and text messages.	The use of text messages significantly improved the clinical measures of BOMP.
Philippot et al., 2005 [74]	Leventhal’s theory. Daily records of the improvement in periodontal symptoms.	At the 1-month follow-up, the experimental group showed smaller scores on all indices as compared with the control group.
Godard et al., 2011 [73]	Single session of MI guided by Leventhal’s theory (15–20′) by two experienced periodontists introduced to the practice of MI.	The test group showed statistically significant improvement compared to the control group.
Stenman et al., 2012 [69] And Stenman et al., 2018 [70]	Single session of MI (20–90′) by a psychologist with extensive experience in MI.	No statistically significant difference between the groups.
Brand et al., 2013 [71]	Single brief session of MI (15–20′) by a trained and experienced counselor in MI.	No statistically significant difference between the groups.
Woelber et al., 2016 [72]	4–5 sessions of MI delivered by dental students trained in MI.	No statistically significant difference between the groups in all the clinical parameters. The test group showed significantly higher interdental cleaning self-efficacy than the control group.
Alcouffe et al., 1988 [75]	Interviewed by a psychologist (50–90′): assessment of their perception of periodontal disease, recovery, prevention, and personal hygiene measures.	Test group: the majority of the patients improved their PI to below 50% after 1 year. Control group: the majority of patients remained stable or worsened.
Jönsson et al., 2006 [76]	4 sessions of Client Self-care Commitment Model (CSCCM) by an experienced dental hygienist	Test group at 3 months: Statistically significant improvement in PI compared to the control group. Statistically significant increase in the use of interdental cleaning. No statistically significant difference in the reduction in PD > 4 mm between the groups.
Jönsson et al., 2009 [77]	5–9 visits of individually tailored oral health educational program based on CBT, using MI, delivered by a trained dental hygienist.	Statistically significant improvement in PI and GI in the test group between both baseline and 3-month follow-up and baseline and 12-month follow-up compared to the control group. Test group reported a higher frequency of daily inter-dental cleaning.
Jönsson et al., 2010 [78]	5–9 visits of individually tailored oral health educational program based on CBT, using MI, delivered by a trained dental hygienist.	Statistically significant improvement in PI and GI in the test group between both baseline and 3-month follow-up and baseline and 12-month follow-up compared to the control group. No group difference for “pocket closure” and reduction in periodontal pocket depth. More individuals in the test group reached a level of treatment success.

Abbreviations: CI, confidence intervals; OR, odds ratio; T, test; C, control; OHI, oral hygiene instructions; MI, motivational interviewing; PD, pocket depth; PPD, probing pocket depth; PI, plaque index; PS, plaque score; FMPS, full mouth plaque score; GI, gingival index; GB, gingival bleeding; BoP, bleeding on probing; BI, bleeding index; CAL, clinical attachment level; HAPA; FF, Finger Floss; NFH, novel floss holder; and MBI, marginal gingival bleeding.

**Table 5:** Quality assessment of the randomized controlled trials based on Cochrane risk-of-bias tool (RoB-2).

	D1	D2	D3	D4	D5	Overall Bias
Williams et al. [65]	HIGH	LOW	LOW	LOW	SOME CONCERN	HIGH
Little et al. [66]	SOME CONCERN	LOW	LOW	HIGH	HIGH	HIGH
Weinstein et al. [67]	HIGH	HIGH	HIGH	HIGH	SOME CONCERN	HIGH
Baab et al. [68]	SOME CONCERN	HIGH	LOW	HIGH	HIGH	HIGH
Stenman et al. [69]	SOME CONCERN	LOW	LOW	LOW	SOME CONCERN	SOME CONCERN
Stenman et al. [70]	HIGH	SOME CONCERN	LOW	LOW	SOME CONCERN	HIGH
Brand et al. [71]	LOW	LOW	LOW	LOW	SOME CONCERN	SOME CONCERN
Woelber et al. [72]	HIGH	HIGH	HIGH	SOME CONCERN	HIGH	HIGH
Godard et al. [73]	LOW	LOW	LOW	HIGH	SOME CONCERN	HIGH
Philippot et al. [74]	SOME CONCERN	HIGH	HIGH	HIGH	SOME CONCERN	HIGH
Alcouffe et al. [75]	HIGH	HIGH	HIGH	HIGH	HIGH	HIGH
Jönsson et al. [76]	HIGH	SOME CONCERN	LOW	LOW	LOW	HIGH
Jönsson et al. [77]	LOW	LOW	LOW	LOW	SOME CONCERN	SOME CONCERN
Jönsson et al. [78]	LOW	LOW	LOW	LOW	SOME CONCERN	SOME CONCERN
Asimakopoulou et al. [79]	HIGH	SOME CONCERN	LOW	HIGH	LOW	HIGH
Araújo et al. [80]	HIGH	HIGH	HIGH	LOW	SOME CONCERN	HIGH

Domains: D1: Bias arising from the randomization process; D2: Bias due to deviations from the intended interventions; D3: Bias due to missing outcome data; D4: Bias in measurement of the outcome; and D5: Bias in selection of the reported result.

**Table 6:** Quality assessment of the non-randomized studies using the Newcastle–Ottawa scale.

	Glavind et al. [82]	Glavind et al. [83]	Glavind et al. [84]	Glavind et al. [81]	Jönsson et al. [85]
Newcastle–Ottawa Assessment criteria:					
Representativeness of the exposed cohort	*	*	*	*	*
Selection of non-exposed cohort	*	*	*	*	*
Ascertainment of exposure					*
Demonstration that the outcome of interest was not present at start of study	*	*	*	*	*
Comparability of cohorts					*
Assessment of outcome	*	*	*	*	*
Was follow-up sufficient	*	*	*		*
Adequacy of follow up	*	*	*	*	*
TOTAL	6	6	6	5	8

## Data Availability

Not applicable.

## Supplementary Materials

The following supporting information can be downloaded at: https://www.mdpi.com/article/10.3390/jcm12062276/s1, Table S1: Excluded full-text articles screened for eligibility with reason for exclusion.

## Abbreviations

OH	oral hygiene
BoP	bleeding on probing
SPT	supportive periodontal therapy
SCMs	social cognition models
CBT	cognitive behavioral therapy
MI	motivational interviewing
PICOS	patient, intervention, control, outcome, and study design
RCTs	randomized controlled clinical trials
NRCTs	non-randomized controlled clinical trials
CI	confidence intervals
OR	odds ratio
NR	not reported
T	test
C	control
OHI	oral hygiene instructions
PPD	probing pocket depth
PD	pocket depth
PI	plaque index
PS	plaque score
FMPS	full mouth plaque score
GI	gingival index
GB	gingival bleeding
BI	bleeding index
BOMP	bleeding on marginal probing
CAL	clinical attachment level
CSCCM	client self-care commitment model
