# Supplementary material for: Behavioral Interventions on Periodontitis Patients to Improve Oral Hygiene: A Systematic Review
Source: J Clin Med. 2023 Mar 15;12(6):2276. doi: 10.3390/jcm12062276 (PMC10058764; doi:10.3390/jcm12062276)
Supplement: Supplementary file 1 [file jcm-12-02276-s001.zip › jcm-2081719-supplementary.pdf]

Table S1. Excluded full-text articles screened for eligibility with reason for exclusion.

**Inappropriate design**
Rayant *et al.* 1980
Chang *et al.* 2019
Kim *et al.* 2020
Ojima *et al.* 2003
Tobias *et al.* 2020
Tedesco *et al.* 1991
Tedesco *et al.* 1993
Jonsson *et al.* 2009a
Kühner *et al.* 1989
**Inappropriate study population**
Schensul *et al.* 2021
Zenthöfer *et al.* 2012
Woelber *et al.* 2015
Ramsay *et al.* 2018
Araújo *et al.* 2020
Barker *et al.* 1994
Al-ak'hali *et al.* 2020
Harnacke *et al.* 2012
Deinzer *et al.* 2016
Garyga *et al.* 2019
Shida *et al.* 2020
Zahid *et al.* 2020
Marshman *et al.* 2019
Kay *et al.* 2019
Lim *et al.* 1996
Araújo *et al.* 2016
Araújo *et al.* 2019
Ziebolz *et al.* 2009
Clarkson *et al.* 2021
Clarkson *et al.* 2013
Stewart *et al.* 1996
Jaedicke *et al.* 2018
**Inappropriate study outcome**
Bajwa *et al.* 2007
Lin *et al.* 2019
**Follow-up < 1 month**
Kakudate *et al.* 2009

Supplementary Table S1 References

S1. Rayant, G.A. and Sheiham, A. (1980), An analysis of factors affecting compliance with tooth-cleaning recommendations. *J Clin Periodontol*. **1980**; 7: 289-299. [CrossRef]

S2. Chang WJ, Lo SY, Kuo CL, Wang YL, Hsiao HC. Development of an intervention tool for precision oral self-care: Personalized and evidence-based practice for patients with periodontal disease. Denis F, ed. *PLoS ONE*. **2019**; 14(11):e0225453. [CrossRef]

S3. Kim JM, Lee WR, Kim JH, Seo JM, Im C. Light-Induced Fluorescence-Based Device and Hybrid Mobile App for Oral Hygiene Management at Home: Development and Usability Study. *JMIR Mhealth Uhealth*. **2020**; 8(10):e17881. [CrossRef]

S4. Ojima M, Hanioka T, Kuboniwa M, Nagata H, Shizukuishi S. Development of Web-based intervention system for periodontal health: a pilot study in the workplace. *Medical Informatics and the Internet in Medicine*. **2003**; 28(4):291-298. [CrossRef]

S5. Tobias G, Spanier AB. Developing a Mobile App (iGAM) to Promote Gingival Health by Professional Monitoring of Dental Selfies: User-Centered Design Approach. *JMIR Mhealth Uhealth*. **2020**; 8(8):e19433. [CrossRef]

S6. Tedesco LA, Keffer MA, Fleck-Kandath C. Self-efficacy, reasoned action, and oral health behavior reports: a social cognitive approach to compliance. *J Behav Med*. **1991**; 14(4):341-355. [CrossRef]

S7. Tedesco LA, Keffer MA, Davis EL, Christersson LA. Self-efficacy and reasoned action: Predicting oral health status and behaviour at one, three, and six month intervals. *Psychology & Health*. **1993**; 8:105-121. [CrossRef]

S8. Jönsson B, Öhrn K, Oscarson N, Lindberg P. The effectiveness of an individually tailored oral health educational programme on oral hygiene behaviour in patients with periodontal disease: a blinded randomized-controlled clinical trial (one-year follow-up). *Journal of Clinical Periodontology*. **2009**; 36(12):1025-1034. [CrossRef]

S9. Kühner MK, Raetzke PB. The Effect of Health Beliefs on the Compliance of Periodontal Patients with Oral Hygiene Instructions. J Periodontol. 1989; 60(1):51-56. [CrossRef]

S10. Schensul J, Reisine S, Salvi A, Ha T, Grady J, Li J. Evaluating mechanisms of change in an oral hygiene improvement trial with older adults. *BMC Oral Health*. **2021**; 21(1):362. [CrossRef]

S11. Zenthöfer A, Dieke R, Dieke A, Wege KC, Rammelsberg P, Hassel AJ. Improving oral hygiene in the long-term care of the elderly-a RCT. *Community Dent Oral Epidemiol*. **2013**; 41(3):261-268. [CrossRef]

S12. Woelber JP, Bienas H, Fabry G, et al. Oral hygiene-related self-efficacy as a predictor of oral hygiene behaviour: a prospective cohort study. *J Clin Periodontol*. **2015**; 42(2):142-149. [CrossRef]

S13. Ramsay CR, Clarkson JE, Duncan A, et al. Improving the Quality of Dentistry (IQuaD): a cluster factorial randomised controlled trial comparing the effectiveness and cost–benefit of oral hygiene advice and/or periodontal instrumentation with routine care for the prevention and management of periodontal disease in dentate adults attending dental primary care. *Health Technol Assess*. **2018**; 22(38):1-144. [CrossRef]

S14. Araújo MR, Alvarez MJ, Godinho CA, Almeida T, Pereira CR. Self-regulation in oral hygiene behaviours in adults with gingivitis: The mediating role of coping planning and action control. *Int J Dent Hyg*. **2020**; 18(2):192-200. [CrossRef]

S15. Barker T. Role of health beliefs in patient compliance with preventive dental advice. *Community Dentistry and Oral Epidemiology*. **1994**; 22(5PT1):327-336. [CrossRef]

S16. Al-ak\'hali MS, Halboub ES, Asiri YM, Asiri AY, Maqbul AA, Khawaji MA. WhatsApp-assisted Oral Health Education and Motivation: A Preliminary Randomized Clinical Trial. *The Journal of Contemporary Dental Practice*. **2020**; 21(8):922-925. [CrossRef]

S17. Harnacke D, Beldoch M, Bohn GH, Seghaoui O, Hegel N, Deinzer R. Oral and Written Instruction of Oral Hygiene: A Randomized Trial. *Journal of Periodontology*. **2012**; 83(10):1206-1212. [CrossRef]

S18. Deinzer R, Harnacke D, Mengel R, Telzer M, Lotzmann U, Wöstmann B. Effectiveness of Computer-Based Training on Toothbrush Skills of Patients Treated With Crowns: A Randomized Controlled Trial. *Journal of Periodontology*. 2016; 87(11):1333-1342. [CrossRef]

S19. Garyga V, Pochelu F, Thivichon-Prince B, et al. GoPerio - impact of a personalized video and an automated two-way text-messaging system in oral hygiene motivation: study protocol for a randomized controlled trial. *Trials*. 2019; 20(1):699. [CrossRef]

S20. Shida H, Okabayashi S, Yoshioka M, et al. Effectiveness of a digital device providing real-time visualized tooth brushing instructions: A randomized controlled trial. Alikhani M, ed. *PLoS ONE*. 2020; 15(6):e0235194. [CrossRef]

S21. Zahid T, Alyafi R, Bantan N, Alzahrani R, Elfirt E. Comparison of Effectiveness of Mobile App versus Conventional Educational Lectures on Oral Hygiene Knowledge and Behavior of High School Students in Saudi Arabia. *PPA*. 2020; Volume 14:1901-1909. [CrossRef]

S22. Marshman Z, Ainsworth H, Chestnutt IG, et al. Brushing RemInder 4 Good oral HealTh (BRIGHT) trial: does an SMS behaviour change programme with a classroom-based session improve the oral health of young people living in deprived areas? A study protocol of a randomised controlled trial. *Trials*. 2019; 20(1):452. [CrossRef]

S23. Kay E, Shou L. A randomised controlled trial of a smartphone application for improving oral hygiene. *Br Dent J*. 2019; 226(7):508-511. [CrossRef]

S24. Lim LP, Davies WIR, Yuen KW, Ma MH. Comparison of modes of oral hygiene instruction in improving gingival health. *J Clin Periodontol*. 1996; 23(7):693-697. [CrossRef]

S25. Araújo MR, Alvarez MJ, Godinho CA, Pereira C. Psychological, behavioral, and clinical effects of intra-oral camera: a randomized control trial on adults with gingivitis. *Community Dent Oral Epidemiol*. 2016; 44(6):523-530. [CrossRef]

S26. Araújo M, Alvarez M, Godinho CA, Roberto MS. An eight-month randomized controlled trial on the use of intra-oral cameras and text messages for gingivitis control among adults. *Int J Dent Hygiene*. 2019; 17(3):202-213. [CrossRef]

S27. Ziebolz D, Herz A, Brunner E, Hornecker E, Mausberg RF. Individual Versus Group Oral Hygiene Instructions for Adults. *Oral Health*. 2009; 7(1):8. [CrossRef]

S28. Clarkson J, Ramsay C, Lamont T, et al. Examining the impact of oral hygiene advice and/or scale and polish on periodontal disease: the IQuaD cluster factorial randomised controlled trial. *Br Dent J*. 2021; 230(4):229-235. [CrossRef]

S29. Clarkson JE, Ramsay CR, Averley P, et al. IQuaD dental trial; improving the quality of dentistry: a multicentre randomised controlled trial comparing oral hygiene advice and periodontal instrumentation for the prevention and management of periodontal disease in dentate adults attending dental primary care. *BMC Oral Health*. 2013; 13:58. [CrossRef]

S30. Stewart JE, Wolfe GR, Maeder L, Hartz GW. Changes in dental knowledge and self-efficacy scores following interventions to change oral hygiene behavior. *Patient Educ Couns.* 1996; 27(3):269-77. [CrossRef]

S31. Jaedicke KM, Bissett SM, Finch T, Thornton J, Preshaw PM. Exploring changes in oral hygiene behaviour in patients with diabetes and periodontal disease: A feasibility study. *Int J Dent Hyg*. **2019**; 17(1):55-63. [CrossRef]

S32. Bajwa A, Watts TLP, Newton JT. Health control beliefs and quality of life considerations before and during periodontal treatment. *Oral Health Prev Dent*. **2007**; 5(2):4. [CrossRef]

S33. Lin JH, Huang YK, Lin KD, Hsu YJ, Huang WF, Huang HL. Randomized Controlled Trial on Effects of a Brief Clinical-Based Intervention Involving Planning Strategy on Self-Care Behaviors in Periodontal Patients in Dental Practice. *IJERPH*. 2019; 16(20):3838. [CrossRef]

S34. Kakudate N, Morita M, Sugai M, Kawanami M. Systematic cognitive behavioral approach for oral hygiene instruction: A short-term study. *Patient Education and Counseling*. 2009; 74(2):191-196. [CrossRef]
